# Tailoring Persuasive Electronic Health Strategies for Older Adults on the Basis of Personal Motivation: Web-Based Survey Study

**DOI:** 10.2196/11759

**Published:** 2019-09-06

**Authors:** Lex van Velsen, Marijke Broekhuis, Stephanie Jansen-Kosterink, Harm op den Akker

**Affiliations:** 1 eHealth Group Roessingh Research and Development Enschede Netherlands; 2 Biomedical Signals and Systems Group University of Twente Enschede Netherlands

**Keywords:** persuasive communication, health communication, software design

## Abstract

**Background:**

Persuasive design, in which the aim is to change attitudes and behaviors by means of technology, is an important aspect of electronic health (eHealth) design. However, selecting the right persuasive feature for an individual is a delicate task and is likely to depend on individual characteristics. Personalization of the persuasive strategy in an eHealth intervention therefore seems to be a promising approach.

**Objective:**

This study aimed to develop a method that allows us to model motivation in older adults with respect to leading a healthy life and a strategy for personalizing the persuasive strategy of an eHealth intervention, based on this user model.

**Methods:**

We deployed a Web-based survey among older adults (aged >60 years) in the Netherlands. In the first part, we administered an adapted version of the revised Sports Motivation Scale (SMS-II) as input for the user models. Then, we provided each participant with a selection of 5 randomly chosen mock-ups (out of a total of 11), each depicting a different persuasive strategy. After showing each strategy, we asked participants how much they appreciated it. The survey was concluded by addressing demographics.

**Results:**

A total of 212 older adults completed the Web-based survey, with a mean age of 68.35 years (SD 5.27 years). Of 212 adults, 45.3% were males (96/212) and 54.7% were female (116/212). Factor analysis did not allow us to replicate the 5-factor structure for motivation, as targeted by the SMS-II. Instead, a 3-factor structure emerged with a total explained variance of 62.79%. These 3 factors are intrinsic motivation, acting to derive satisfaction from the behavior itself (5 items; Cronbach alpha=.90); external regulation, acting because of externally controlled rewards or punishments (4 items; Cronbach alpha=.83); and a-motivation, a situation where there is a lack of intention to act (2 items; *r*=0.50; *P*<.001). Persuasive strategies were appreciated differently, depending on the type of personal motivation. In some cases, demographics played a role.

**Conclusions:**

The personal type of motivation of older adults (intrinsic, externally regulated, and/or a-motivation), combined with their educational level or living situation, affects an individual’s like or dislike for a persuasive eHealth feature. We provide a practical approach for profiling older adults as well as an overview of which persuasive features should or should not be provided to each profile. Future research should take into account the coexistence of multiple types of motivation within an individual and the presence of a-motivation.

## Introduction

### Background

In recent years, persuasive design has become an integral part of electronic health (eHealth). Persuasive technology aims to change people’s attitudes and behaviors [1] and can be an extremely valuable instrument in increasing patient adherence to Web-based health interventions [2,3], improving self-management abilities [4], and more positive perceptions of usability [5]. To facilitate the design of persuasive systems, a wide range of features have been listed (most notably in a study by Oinas-Kukkonen and Harjumaa [6]). These features include rewarding target behavior, creating trustworthiness, and applying personalized content or services. However, designing a persuasive eHealth intervention is not an easy task of inserting as many persuasive elements as possible. Instead, when combining specific principles, their workings can reinforce one another (eg, combining social learning and comparison), whereas combining too many persuasive features diminishes the effect of the intervention [7]. Choosing the optimal set of persuasive features is a delicate task and might very well depend on the individual end user’s characteristics. Tailoring the set of persuasive features a person has to his or her avail, therefore, seems to be a very promising approach [8].

Tailoring, as defined in a study by Hawkins et al, pertains to “any of a number of methods for creating communications individualized for their receivers, with the expectation that this individualization will lead to larger intended effects of these communications” [9]. Although the term is often interchangeably used with *personalization*, we use the term *tailoring* as an umbrella term to cover various specific concepts, such as feedback, context awareness, or user targeting, as defined in a study by op den Akker et al [10]. Research in and applications of tailoring typically focus on specific communications. The Handheld Exercise Agent from Bickmore et al is a good example of this, where tailoring is adopted by providing feedback on the user’s personal physical activity and using context-aware features to provide assistive messages in certain situations [11]. Other studies have delved into using machine learning to choose opportune moments for health messages [12] or the use of different framing techniques to increase their intended effect [13]. However, irrespective of whether the technology used is basic or advanced, the principle of the process always remains the same—one must *measure* one or more characteristics of the user (user modeling), *reason* on, and adapt the specific communication to provide and *reach* the individual with the specific outcome.

Studies that aimed to identify the most effective persuasive eHealth tactics for specific user groups have mainly tried to single out a set of features that work best for a given population. For example, Karppinen et al [14] identified a subset of persuasive features that work specifically well for an eHealth intervention, aimed at persons at risk for or suffering from metabolic syndrome. Similarly, Smith et al [15] uncovered a set of features that have the best effect on melanoma checking using a digital intervention. However, they also conclude that personality traits should be taken into account when selecting the most suitable persuasion strategy for an individual. In their context, emotional stability appeared to be a decisive factor for this selection. Other research has reached similar conclusions [16,17]. A promising way to tailor a persuasive strategy, which has not been explored before, is to use an end user’s motivation to work on his or her health as the main decisive factor.

### Objectives

In this paper, we report on a study that aims to define a strategy for tailoring the set of persuasive features for older adults in an eHealth intervention, aimed at promoting a healthy lifestyle. The basis of this tailoring strategy is a user model in which the individual older adult’s motivation to lead a healthy life is modeled. Individual motivation has been identified as a crucial factor for explaining older adults’ physical activity levels [18], social participation [19], and cognitive health [20]. Therefore, in the first part of this study, we develop a method for modeling older adults’ motivation to adopt a healthy lifestyle. In the second part of this study, we elicit the preferred persuasive eHealth strategies for each motivational profile that was identified.

### Theoretical Background

In the psychological literature, there are ample theories and methods to classify different types of motivation. Self-determination theory (SDT), a prominent motivational theory, proposes that the level of autonomy and control [21] influences the level of motivation for a specific action or behavior. Autonomous motivation means that the individual voluntarily performs a behavior because he or she enjoys the activity or finds it interesting. Controlled motivation means that an individual performs a behavior because of external rewards or (social) pressure. SDT describes a continuum of autonomy and control. People can become more or less autonomous or controlled in their motivation. According to SDT, there are 6 types of motivation [21]: (1) *intrinsic motivation*, where one acts because one derives satisfaction from the behavior itself; (2) *extrinsic motivation* *—* *integrated regulation*, where one acts because the behavior is in line with one’s life goals, objectives, and needs; (3) *extrinsic motivation* *—* *identified regulation*, where one acts because something is considered personally important and worthwhile; (4) *extrinsic motivation* *—* *introjected regulation*, where one acts to feel worthy, out of guilt, or to avoid shame; (5) *extrinsic motivation* *—* *external regulation*, where externally controlled rewards or punishments direct behavior; and (6) *a-motivation*, a situation where there is a lack of intention to act.

Integrated, identified, introjected, and external regulation are all subtypes of extrinsic motivation but differ in the level of autonomy and control. Nonetheless, their common denominator is that a person behaves in a certain way because it leads to a desired outcome, such as receiving a reward, feeling less guilty, or acting in accordance with one’s personal values. People who are intrinsically motivated perform the behavior because they like the activity itself [22]. In contrast, although both intrinsic and extrinsic motivations imply the intention to act, a-motivation implies a lack of intention. More fine-grained approaches toward understanding motivation and measurement instruments for assessing this motivation have been developed for different contexts (eg, motivation to exercise [23]). At the moment of writing, however, no methods are available for classifying different kinds of motivation among older adults to lead a healthy lifestyle.

As we mentioned before, a person’s type of motivation can play a role when selecting the most suitable persuasive strategy for an individual. This assertion has been studied mostly in the context of promoting exercise and physical activity although not necessarily by means of eHealth interventions. Ingledew and Markland [24] found that, in general, different types of motivation can be associated with different motives for physical exercise and that persuasive strategies should comply with these motives. Kaptein et al describe how they developed personalized persuasive systems using personal persuasion profiles and demonstrate how such systems can persuade people to eat healthier or be more physically active [25]. De Vries et al [26] used the transtheoretical model (which discerns different stages of change for an individual) to provide personalized encouragement for physical exercise through digital services. In another study, De Vries et al [27] found that the Big Five classification can predict the appreciation of different motivational messages, sometimes moderated by the demographic gender. The role of demographics in explaining older adults’ health-related behaviors and their motivations for living a healthy lifestyle has been generally acknowledged [28,29].

### Research Model and Hypotheses

**Figure 1:**
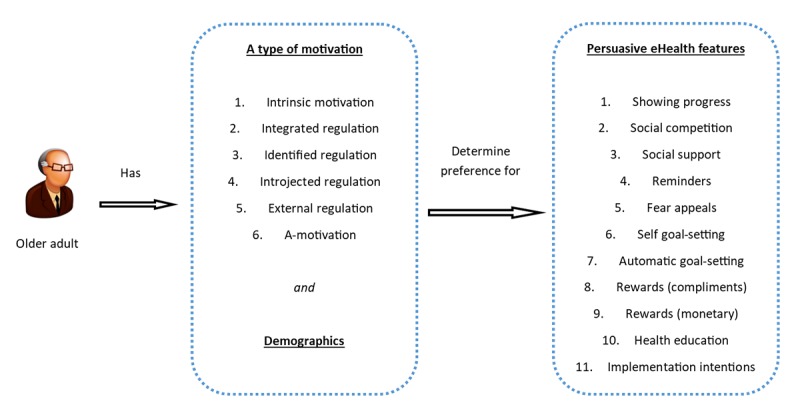
Research model. eHealth: electronic health.

On the basis of the theoretical background, we created a research model, as depicted in Figure 1.

The research model makes several assumptions. First, it posits that an individual older adult has a specific type of motivation to work on his or her health. This assumption has been tested numerous times for different health-related contexts, with the SDT as the basis for conceptualizing personal motivation [30].

Hypothesis 1: An older adult has a personal motivation for working on his or her health that can be classified as intrinsic motivation, integrated regulation, identified regulation, introjected regulation, external regulation, or a-motivation.

Next, we hypothesize that the persuasive features that an older adult appreciates in an eHealth service can be explained by this type of motivation. Previously, it was found, when motivating people to comply with a physical exercise regime in an offline setting, that persuasive strategies are more effective when tailored to an individual’s motivation [24].

Hypothesis 2: Each type of motivation is linked to a unique set of highly appreciated persuasive features.

Finally, we hypothesize that, besides an individual’s motivation, demographic factors also predict appreciation of a persuasive feature. This assertion has been scarcely studied before. Age has been found to play a role in the appreciation of persuasive electronic service features in the context of energy saving [31]. Nonetheless, demographics do explain to an important extent to what degree an eHealth intervention is adopted [32,33]. Therefore, we expect them to play a similar role in the formation of appreciation of persuasive features.

Hypothesis 3: An older adult’s demographic traits (age, gender, education level, and living situation) predict his or her appreciation of a persuasive feature within an eHealth service.

## Methods

### Overview

We conducted a Web-based survey among older adults in the Netherlands, which consisted of 3 parts. First, we collected data to test a user profiling method for classifying older adults’ motivation to adopt a healthy lifestyle. Second, we provided each participant with a set of persuasive eHealth strategies and asked them to indicate their appreciation for each feature. Finally, we assessed each participant’s demographics. The survey was provided through a Web-based survey environment.

### User Profiling

To model personal motivation, we used the revised Sports Motivation Scale (SMS-II) [34], a validated instrument for assessing sports motivation; a domain closely related to adopting a healthy lifestyle. The survey is based on the SDT [21] and clusters people on the 6 types of motivation of the SDT (intrinsic motivation, integrated regulation, identified regulation, introjected regulation, external regulation, and a-motivation). We adapted the questionnaire to the needs of this specific context. Each type of motivation, or construct, was assessed via 3 statements, accompanied by a 7-point Likert scale (ranging from very much disagree [1] to very much agree [7]).

### Preferred Persuasive Features

We assessed the appreciation of 11 persuasive features or strategies. The features in this list were selected based on their popularity in existing eHealth interventions.

1. Showing progress: displaying how many recent activities have helped to reach specific health goals.
2. Social competition: showing how healthy your behavior is in relation to peers, in the form of leaderboards.
3. Social support: connecting peers so that they can motivate each other to reach health goals.
4. Reminders: reminding individuals to act healthy or to do exercises by means of pop-ups on, for example, mobile devices.
5. Fear appeals: instilling fear about the current lifestyle of a person by explicating the negative consequences.
6. Self-goal setting: allowing a person to set health goals him/herself (in the mock-up visualized by setting weekly step goal).
7. Automatic goal setting: automated health goal setting for a person (in the mock-up displayed by setting a weekly step goal, based on past performance).
8. Rewards—compliments: complimenting a person on reaching health goals (in the form of badges).
9. Rewards—monetary: awarding points when reaching health goals that can be used at a Web-based shop.
10. Health education: educating persons about the benefits of healthy behavior and the way in which the body reacts to this behavior.
11. Implementation intentions: providing the possibility to plan healthy activities (eg, walking and yoga) in a Web-based, weekly planner.

**Figure 2:**
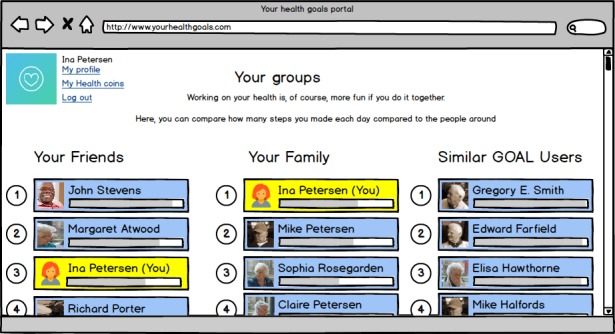
Mock-up of the persuasive feature "social competition".

**Figure 3:**
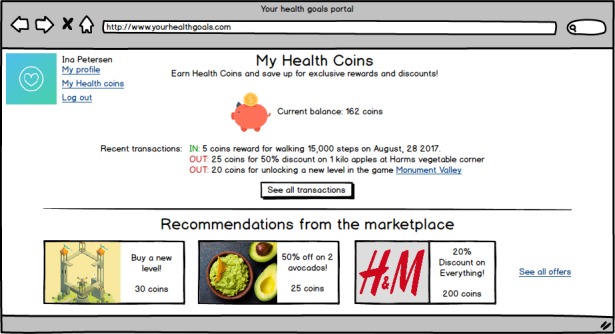
Mock-up of the persuasive feature "monetary rewards".

Each persuasive feature was presented to the participant in the form of a simple digital mock-up, which was created in Balsamiq(Balsamiq Studios). Figures 2 and 3 show two of these mock-ups (social competition and monetary rewards) as examples. Multimedia Appendix 1 gives an overview of all mock-ups. To keep the length of the survey acceptable, we asked each participant to rate 5 of the 11 persuasive features available in total. These 5 features were randomly selected and presented. Each feature was accompanied by a statement (This [information/feature] would motivate me to work on my health) and a 7-point Likert scale ranging from very much disagree [1] to very much agree [7]).

### Demographics

For each participant, we assessed gender, age, educational level (none, elementary school, basic level high school, vocational education, high level high school, and college/university), living situation (living together/married, single, and other), and self-reported physical activity level (by asking them to choose 1 of 5 options: not sporting, not sporting but thinking about beginning, sporting <2.5 hours a week, sporting >2.5 hours a week in the last 6 months, or sporting >2.5 hours a week for more than 6 months). Then we questioned participants’ self-reported health by asking participants to rate their health on each of the aspects of health, according to the Positive Health approach [35] (bodily functions, mental functions and perception, spiritual and existential health, quality of life, social and societal participation, and daily functioning), using a 10-point rating scale (ranging from 1 [very poor] to 10 [excellent]). Examples of these statements are as follows: How healthy do you rate your own body? Do you feel fit? Do you have pain somewhere? And can you sleep and eat well? (for physical health), or What do you think of your social life? Do you have enough friends? Do you have others to do fun things with? Do you get help when you need it? And do you have the feeling of belonging somewhere? (for social and societal participation). Finally, we assessed health literacy using the health literacy scale by Chew et al [36], consisting of 3 statements and accompanied by a 6-point Likert scale (ranging from 1 [negative] to 6 [positive]). These statements are as follows: How often do you have problems learning about your medical condition because of difficulty understanding written information? How confident are you filling out medical forms by yourself? How often do you have someone help you read hospital materials?

### Recruitment

Participants were eligible if they were aged 60 years or older. We recruited participants through a Dutch panel of older adults that indicated they were interested in participating in research on the topic of eHealth. In addition, we used snowball sampling through social media and personal connections.

### Statistical Analyses

All data were analyzed in SPSS 19.0 (SPSS Inc). Descriptive statistics were calculated for demographic variables (percentages, means, and SDs). To determine the suitability of the SMS-II for factor analysis, we assessed interitem correlations, the Kaiser-Meyer-Olkin measure for sampling adequacy, and the Bartlett test of sphericity, and we focused on the diagonals of the anti-image correlation matrix and item communalities. To uncover the factor structure underlying the SMS-II data, we conducted a factor analysis with principal axis factoring and oblimin rotation. The reliability of the resulting factors was assessed by assessing item-to-total correlations and the factor’s skewness, kurtosis, and Cronbach alpha (in case a factor had more than 2 items) or by calculating Pearson correlation (in case a factor consisted of 2 items). Distribution of the scores for the different motivational factors was made insightful by means of boxplots, whereas overlap among the factors was displayed using a scatterplot and supported by Pearson correlations. To determine which type of motivation predicts the appreciation of the 11 different persuasive features, we first checked the distribution of these appreciation scores for normality (which they did). Second, we assessed mean appreciation scores and SDs and calculated correlation scores between types of motivation and the appreciation of persuasive features. Finally, we conducted linear regression analyses. At first, we created models wherein appreciation predicts one type of motivation. If significant, we ran a second analysis where we also included the demographics sex, age, living situation, and education as main effects using the Enter method. For this analysis, the variable *education* was split into low and high education, and for the variable *living situation*, the answering option *other* was classified as missing variable (which only occurred once among 212 cases) so that we only had 2 answering options. These changes enabled us to include these variables in regression analyses.

### Ethics

Digital informed consent was obtained from each respondent. The nature of this general internet-based survey among healthy volunteers from the general population does not require formal medical ethical approval according to Dutch law.

## Results

### Demographics

Data collection resulted in 212 valid participants. Of 212 participants, 45.3% (96/212) was male, and 54.7% (116/212) was female. Their ages ranged from 60 to 93 years (mean 68.35 [SD 5.27]). Their educational background consisted of elementary school (1.4%, 3/212), lower vocational education (17.5%, 37/212), vocational education (21.7%, 46/212), high school (14.2%, 30/212), or higher vocational education or university (45.3%, 96/212). Most participants lived together with spouse (79.2%, 168/212). Others lived alone (20.3%, 43/212) or had other living arrangements (0.5%, 1/212).

The participants rated their health literacy as high with a mean of 4.91 (SD 0.63) on a 6-point scale. With regard to their current physical activity behavior, 9/212 participants stated that they did not exercise or compete in sports and were not planning to do so (4.2%); 6/212 participants were not doing this but were thinking about starting (2.8%). Most participants were already physically active. In addition, 45/212 participants (21.2%) exercised or competed in sports, but not on a regular basis (<2.5 hours a week), 44/212 participants (20.8%) exercised or competed in sports regularly between the last 6 months and now (>2.5 hours a week), and 108/212 participants (50.9%) exercised or competed in sports regularly for a period longer than 6 months (>2.5 hours a week). The participants rated their health quite high on different health dimensions:

- Bodily functions: mean 7.27 (SD 1.46)
- Mental functions and perception: mean 7.93 (SD 1.10)
- Spiritual/existential dimension: mean 8.09 (SD 1.15)
- Quality of life: mean 8.04 (SD 1.14)
- Social and societal participation: mean 7.88 (SD 1.38)
- Daily functioning: mean 8.55 (SD 1.02)

### User Profiling

As a first step in developing the measurement model for motivation to live a healthy life (and hence, the user profiling method), we examined the factorability of the 18 measurement items for motivation. All items correlated with at least one other item, with a coefficient of 0.3. Next, the Kaiser-Meyer-Olkin measure for sampling adequacy was 0.89, whereas the Bartlett test of sphericity was significant (χ^2^_153_=2177.9; *P*<.001). The diagonals of the anti-image correlation matrix were also all over 0.6. All item communalities were larger than 0.3. These results indicate that a factor analysis can be conducted with these 18 items.

We conducted a factor analysis with principal axis factoring and oblimin rotation. Initial eigenvalues suggested 3 factors, with an explained variance of 39.67%, 15.38%, and 7.74%, respectively. Total explained variance for these 3 factors was 62.79%. Table 1 displays the item factors loadings and communalities.

The results of the factor analysis show that the 6-factor structure of the SMS-II could not be replicated for a population of older adults when adapted to the context of healthy living (and not sports, as was the original focus of the survey instrument). However, a 3-factor structure became apparent. The first factor, which we call *intrinsic motivation,* focuses on leading a healthy life for personal reasons (eg, because an older adult likes it or wants to develop himself or herself this way). This factor comprised SMS-II items that assess intrinsic motivation and 2 items that, originally, assessed identified regulation but also focus on the self (to develop personal strong suits and develop other sides of myself). The second factor consists of the items that assessed external regulation in the original SMS-II, supplemented by an item from the a-motivation scale that also focuses on external rewards (to get compliments from others). Hence, we retain the name *external regulation*. The third and final factor is *a-motivation*, which consists of the two remaining a-motivation items.

**Table 1:** Factor loadings and communalities (factor loadings <0.3 are suppressed).

Item		Factor			Communality
		1	2	3	
**Intrinsic motivation**					
	Because I like to learn more about healthy living	0.79	—	—	0.60
	Because I like to discover new ways to lead a healthier life	0.88	—	—	0.73
	Because I think it’s very interesting to learn how to live a healthier life	0.82	—	—	0.62
**Integrated regulation**					
	Because by living healthy I show who I am	0.40	0.50	—	0.61
	Because living healthy is an important part of my life	0.52	—	−0.43	0.67
	Because I think it’s very important to live a healthy life	0.43	—	−0.47	0.55
**Identified regulation**					
	Because I chose myself to live a healthier life in order to develop myself	0.59	—	—	0.52
	Because I think it is a good way to develop my strong suits	0.70	—	—	0.63
	Because I think it’s one of the best ways to develop other sides of myself	0.81	—	—	0.69
**Introjected regulation**					
	Because I would feel bad if I didn’t make time for that	0.45	0.39	—	0.53
	Because I would think I am worth little if I did not lead a healthy life	—	0.43	—	0.34
	Because I would feel better if I lead a healthy life	0.57	—	−0.42	0.63
**External regulation**					
	Because the people that are important to me would be angry at me if I didn’t	—	0.71	—	0.45
	Because I would then be appreciated by the people I know	—	0.65	—	0.57
	Because I think other would disapprove of me if I didn’t	—	0.88	—	0.58
**A-motivation**					
	I used to have good reasons to live a healthy life, but lately I’m doubting whether or not to continue with that	—	—	0.61	0.32
	So that I get compliments from others	—	0.76	—	0.61
	I don’t think that living a healthy life really is something for me	—	—	0.64	0.37

Table 2 displays the reliability scores for the emerging measurement scales for intrinsic motivation and external regulation. As the third factor comprised only 2 items, these metrics do not apply for this scale. The correlation between the two items (“I used to have good reasons to live a healthy life, but lately I’m doubting whether or not to continue with that” and “I don’t think that living a healthy life really is something for me”) was significant (*r*=0.50; *P*<.001).

**Figure 4:**
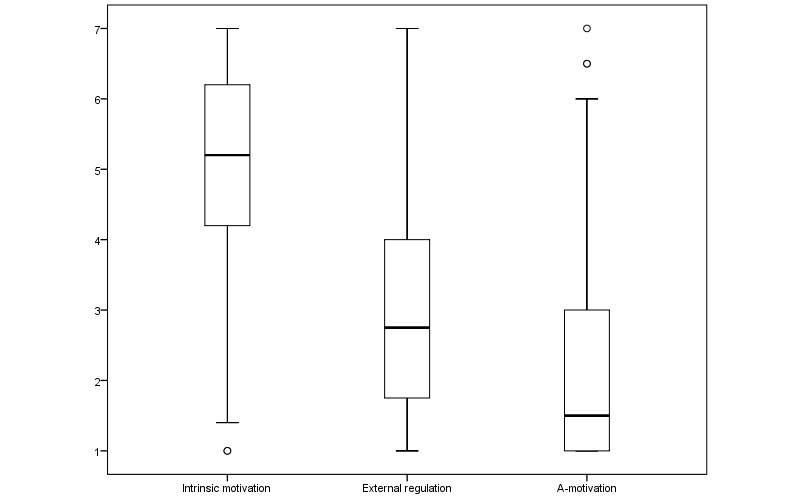
Boxplots of the scores on appreciation for different types of motivation.

Figure 4 displays 3 boxplots of the score distributions for the different types of motivation in our sample. It shows that, on average, participants scored quite high on intrinsic motivation, but there is a wide range in scores. For external regulation, we also see this wide range; but, in general, participants scored below average. With respect to a-motivation, finally, low scores were observed overall, with a small group of participants who scored high.

**Figure 5:**
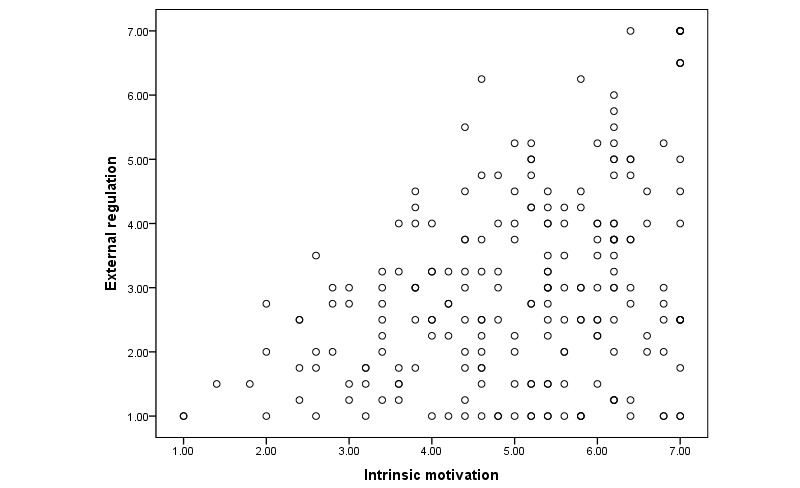
Scatterplot for scores on intrinsic motivation and external regulation.

Furthermore, we created a scatterplot for intrinsic motivation and external regulation (Figure 5) to show overlap between the two types of motivation. It shows that there is some overlap between the two types of motivation. Participants who were intrinsically motivated sometimes also displayed a high degree of external regulation. It was also possible to be intrinsically motivated only. Calculation of the Pearson correlation between these two factors confirms this (*r*=0.38; *P*<.001).

These results partly support our first hypothesis: an older adult has a personal motivation for working on his or her health that can be classified as intrinsic motivation, integrated regulation, identified regulation, introjected regulation, external regulation, or a-motivation. We found that an older adult can be classified as being intrinsically motivated, externally regulated, and a-motivated. However, the classifications are not mutually exclusive.

**Table 2:** Reliability of the measurement scales for intrinsic motivation and external regulation.

Item			Item-to-total correlation		Skewness		Kurtosis		Cronbach alpha
**Intrinsic motivation**					−0.61		−0.22		.90
	Because I like to learn more about healthy living	0.71							
	Because I like to discover new ways to lead a healthier life	0.79							
	Because I think it’s very interesting to learn how to live a healthier life	0.74							
	Because I think it is a good way to develop my strong suits	0.70							
	Because I think it’s one of the best ways to develop other sides of myself	0.79							
**External regulation**					0.70		0.00		.83
	Because the people that are important to me would be angry at me if I didn’t	0.57							
	Because I would then be appreciated by the people I know	0.59							
	Because I think other would disapprove of me if I didn’t	0.72							
	So that I get compliments from others	0.74							

### Appreciation of Persuasive Features

We asked the participants to rate their appreciation for 5 of 11 persuasive features. Table 3 displays how many participants were presented each feature and how they appreciated them. It shows that the appreciations were neutral or positive, whereby fear appeals, monetary rewards, and implementation intentions were appreciated best. In all cases, SDs were high, indicating that participants’ appreciations were diverse.

Furthermore, we assessed the correlations among the different types of motivation on the one hand and the appreciation of persuasive features on the other. The results are presented in Table 4. It shows that intrinsic motivation is correlated to the appreciation of all persuasive features, external regulation to some, and a-motivation to none.

**Table 3:** General appreciation of 11 persuasive features.

Persuasive feature	N	Appreciation, mean (SD)
1. Showing progress	105	3.34 (2.08)
2. Social competition	89	4.14 (1.79)
3. Social support	106	3.92 (1.92)
4. Reminders	106	3.61 (2.09)
5. Fear appeals	101	4.63 (1.99)
6. Self-goal setting	98	3.89 (2.07)
7. Automatic goal setting	109	4.06 (1.88)
8. Rewards—compliments	89	3.40 (1.98)
9. Rewards—monetary	100	4.75 (1.78)
10. Health education	88	3.59 (2.06)
11. Implementation intentions	94	4.40 (1.76)

**Table 4:** Correlations between types of motivation and appreciation of persuasive features.

Types of motivation	Persuasive feature										
	1	2	3	4	5	6	7	8	9	10	11
Intrinsic motivation	0.42^a^	0.47^a^	0.35^a^	0.25^b^	0.38^a^	0.62^a^	0.44^a^	0.42^a^	0.35^a^	0.37^a^	0.37^a^
External regulation	0.37^a^	0.26^c^	0.17	0.29^b^	0.22^c^	0.33^b^	0.26^b^	0.41^a^	0.17	0.46^a^	0.12
A-motivation	0.01	0.05	0.08	−0.01	0.13	0.06	0.02	0.07	−0.02	0.09	0.16

^a^*P*<.001.

^b^*P*<.01.

^c^*P*<.05.

To determine which type of motivation predicts the appreciation of the 11 different persuasive features, we conducted different sets of linear regression analyses: first, assessing the influence of the type of motivation on the appreciation of a feature; second, assessing the role that demographics play (for results, see Table 5). To preserve a legible overview, we only report significant results for the analyses of demographics.

The results show that being intrinsically motivated positively affected the appreciation of all persuasive features, most heavily showing progress, social competition, self-goal setting, automatic goal setting, and rewards in the form of compliments. Being externally regulated positively affected the appreciation of all persuasive features, except for monetary rewards and implementation intentions. The strongest influences were found for the features showing progress, self-goal setting, rewards in the form of compliments, and health education. A-motivation, as the third motivational style, did not affect the appreciation of any of the persuasive features. Finally, with respect to demographics, we found that health education was better appreciated by participants with a lower education and who are either intrinsically motivated or externally regulated. Intrinsically motivated participants who lived alone appreciated implementation intentions better. Gender and age did not play a significant role.

These results partly support our second hypothesis (each type of motivation is linked to a unique set of highly appreciated persuasive features). Appreciation of all the persuasive features that we studied was affected by either intrinsic motivation and/or external regulation. A-motivation did not affect the appreciation of any persuasive feature. There was a distinction between the features that were highly appreciated by people with high intrinsic motivation on the one hand and high external regulation on the other. Finally, our third hypothesis (an older adult’s demographic traits [age, gender, education level, and living situation] predict his or her appreciation of a persuasive feature within an eHealth service) is partly supported by our analyses. Education affected the appreciation of health education (for both the intrinsically motivated and externally regulated persons), whereas participants who had high intrinsic motivation and lived alone appreciated implementation intentions higher.

**Table 5:** Results of regression analyses.

Persuasive feature		Predictor	Beta	*t* test (*df*)	*P* value	*R* ^2^
**Showing progress**						
	1	Intrinsic motivation	.55	6.16 (90)	<.001	0.30
	2	External regulation	.42	4.39 (90)	<.001	0.18
	3	A-motivation	−.04	−0.40 (90)	NS^a^	0.00
**Social competition**						
	1	Intrinsic motivation	.49	5.05 (79)	<.001	0.24
	2	External regulation	.29	2.64 (79)	.010	0.08
	3	A-motivation	.04	0.39 (79)	NS	0.00
**Social support**						
	1	Intrinsic motivation	.35	3.61 (91)	.001	0.13
	2	External regulation	.25	2.44 (91)	.017	0.06
	3	A-motivation	.16	1.51 (91)	NS	0.02
**Reminders**						
	1	Intrinsic motivation	.27	2.60 (84)	.011	0.07
	2	External regulation	.32	3.09 (84)	.003	0.10
	3	A-motivation	−.03	−0.25 (84)	NS	0.00
**Fear appeals**						
	1	Intrinsic motivation	.36	3.46 (83)	.001	0.13
	2	External regulation	.25	2.39 (83)	.019	0.06
	3	A-motivation	.15	1.36 (83)	NS	0.02
**Self-goal setting**						
	1	Intrinsic motivation	.64	7.45 (80)	<.001	0.41
	2	External regulation	.40	3.86 (80)	<.001	0.16
	3	A-motivation	.08	0.74 (80)	NS	0.01
**Automatic goal setting**						
	1	Intrinsic motivation	.43	4.56 (91)	<.001	0.19
	2	External regulation	.34	3.44 (91)	.001	0.12
	3	A-motivation	.05	0.51 (91)	NS	0.00
**Rewards** **—** **compliments**						
	1	Intrinsic motivation	.42	3.91 (73)	<.001	0.17
	2	External regulation	.40	3.74 (73)	<.001	0.16
	3	A-motivation	.06	0.52 (73)	NS	0.00
**Rewards** **—** **monetary**						
	1	Intrinsic motivation	.36	3.60 (87)	.001	0.13
	2	External regulation	.18	1.71 (87)	NS	0.03
	3	A-motivation	.00	−0.02 (87)	NS	0.00
**Health education**						
	1	Intrinsic motivation	.33	3.55 (85)	.001	0.26
		Education	−.36	−3.82 (85)	<.001	
	2	External regulation	.43	4.86 (85)	<.001	0.34
		Education	−.36	−4.04 (85)	<.001	
	3	A-motivation	.07	0.63 (75)	NS	0.01
**Implementation intentions**						
	1	Intrinsic motivation	.35	3.76 (91)	<.001	0.22
		Living situation	−.29	−3.07 (91)	.003	
	2	External regulation	.21	1.89 (80)	NS	0.04
	3	A-motivation	.18	1.66 (80)	NS	0.03

^a^NS: nonsignificant.

## Discussion

### Principal Findings

The use of persuasive features in eHealth services to improve the uptake of health advice has become very popular in recent years. This study shows that different types of older adults prefer different persuasive features when improving their lifestyle by an eHealth service. To profile older adults, so that the offer of persuasive features can be tailored to the individual, we tried to classify older adults’ motivation in this context. For this, we used the SMS-II. However, in this context, we were unable to replicate the classification scheme, as posited by the SDT. Rather, our analysis showed that older adults are intrinsically motivated to work on their health (they derive satisfaction from the behavior itself), externally regulated (they act because of rewards or punishments), or a-motivated (there is a general lack of intention to act). It is also possible to have multiple types of motivation. You can find the final survey for classifying older adults in Multimedia Appendix 2. Most participants could be classified as intrinsically motivated, and smaller groups were externally regulated or a-motivated. Interestingly, we found a relationship between being intrinsically motivated and externally regulated; the two seem to go hand in hand quite regularly.

The appreciation of different persuasive features that aim to motivate older adults to adopt a healthy lifestyle can, so we found, be explained by their motivation. Being intrinsically motivated turned out to be a precursor for appreciating all persuasive features that we tested. Being externally regulated explained appreciation of most persuasive features. By looking at the regression weights and explained variance for each persuasive feature, we can conclude that older adults with a high degree of intrinsic motivation are best served by means of showing progress, social competition, self-goal setting, automatic goal setting, and rewards in the form of compliments. Older adults that are externally regulated would be served best by offering them self-goal setting, rewards in the form of compliments, and health education (especially if they are lower educated).

Finally, being a-motivated turned out to have no effect on the appreciation of persuasive features. This might suggest that older adults who are a-motivated are not in a situation in which they are influenced by persuasive features. Instead, different interventions or strategies need to be installed to get them into a stage of being motivated.

### Comparison With Prior Work

The growing interest in persuasive eHealth design has mainly approached the use of persuasive features as a one-size-fits-all solution, which is strange, as personalization is one of the recommendations in the seminal overview of persuasive features by Oinas-Kukkonen [6]. Only recently have some studies focused on the use of personalized approaches toward offering persuasive eHealth features [15,16]. This study, however, is the first to use the end user’s motivation to act healthy as a main component in the user model and, thus, as a decisive factor for tailoring the persuasive approach.

Contrary to previous work, which has focused on modeling motivation and acting on these models, we have also taken into account a situation wherein a person is a-motivated. As it turned out, this is a specific group of people with a specific stance toward persuasive technology. In general, methods and strategies specifically designed for engaging this group of people are scarce [37]. A-motivation stems from not perceiving the benefits of the activity [38], not believing the activity will lead to certain outcomes [39], or not feeling adequately skilled for the activity [40]. Studies that analyze behavioral reasons for physical activity among different motivation clusters often do not consider a-motivation [41-43], as it entails an absence of intentionality [22]. Motivating this group of people could be done by means of motivational interviewing or changing the a-motivated person’s context (eg, removing places for smoking from the workplace) [37]. Both approaches, however, seem to lie outside the realm of eHealth services, and it might well be the case that for an a-motivated person to use persuasive technology, more intensive, offline types of coaching or transformation need to be applied.

Previous research has treated the different motivation profiles as orthogonal constructs. Our analyses uncovered, however, that multiple types of motivation can exist within one individual (in our case, intrinsic motivation and external regulation mostly). Only recently have researchers recognized that different types of motivation can co-occur [44,45]. Future studies and persuasive eHealth technologies should take into account that a person can be motivated in multiple ways and that persuasive design should be tailored toward this situation. As such, the results of this study support the use of persuasion profiles, in which each type of an individual’s motivation is scored on a preset scale [25].

### Limitations

The operationalization of the 11 different persuasive strategies that we put to the test in this study was done by means of creating low-fidelity mock-ups. Developing high-fidelity prototypes that would allow for end user interaction would have provided stronger stimulus material. On the other hand, it would not have allowed us to explore such a broad range of persuasive strategies, as it would drastically increase the time needed to complete the survey.

Providing personalized, persuasive eHealth interventions should primarily result in health gains (eg, a healthier diet and less time spent sedentary). We used end-user appreciation as the sole indicator of the success of a persuasive strategy. This was done, as it allowed us to provide and test a set of persuasive strategies within a very short time frame. Future studies should take a longitudinal approach to assess this effect. At first, a suitable personalization strategy should be selected based on a model of the individual participant. Then, the participant should be allowed to interact with the personalized eHealth intervention over time so that the intended health effects can occur. Only at this time can the full effect of personalized persuasion be assessed.

In this study, we focused on older adults (aged >60 years) and their motivation to adopt a healthy lifestyle. Next, during recruitment, we used a panel of older adults that showed an interest in eHealth. This led to an overrepresentation of highly educated participants. This might have accounted for the high number of intrinsically motivated participants. In a sample with more lower educated and/or people with less interest in eHealth, the distributions of participants over the different motivational classifications might be somewhat different. However, we do think that for the modeling of motivation and analyses of the relationships between motivational type and the appreciation of different features, this limitation will have no or a very marginal effect. Of course, generalization of these results to other age groups should be done with caution.

### Conclusions

Older adults can be classified as being intrinsically motivated, externally regulated, and/or a-motivated when it comes to working on their health. This study provides a set of survey items that can be used to model each type of motivation for an individual and shows which persuasive features can be used best to engage an older adult in working on his or her health. The fact that we found that different types of motivation can coexist within an individual is contradictory to previous research, which has treated different types of motivation as orthogonal constructs. Next, a-motivation is never considered while designing persuasive eHealth technology. This study has shown that we should take this type of motivation into account. We hope that these lessons will further mature the growing field of persuasive technology and eHealth design.

## Appendix

Multimedia Appendix 1 Mock-ups of persuasive features.

Multimedia Appendix 2 Final survey for classifying older adults.

## Abbreviations

eHealth: electronic health
SDT: self-determination theory
SMS-II: revised Sports Motivation Scale
