# Supplementary material for: Tailoring Persuasive Electronic Health Strategies for Older Adults on the Basis of Personal Motivation: Web-Based Survey Study
Source: J Med Internet Res. 2019 Sep 6;21(9):e11759. doi: 10.2196/11759 (PMC6788334; doi:10.2196/11759)

Multimedia Appendix 1:

Mock-ups of persuasive features

Feature 1. Showing progress

On Yourhealthgoals.com we can inform you about the progress you are making while working on your health. What are your goals? And how far did you progress in reaching these goals?

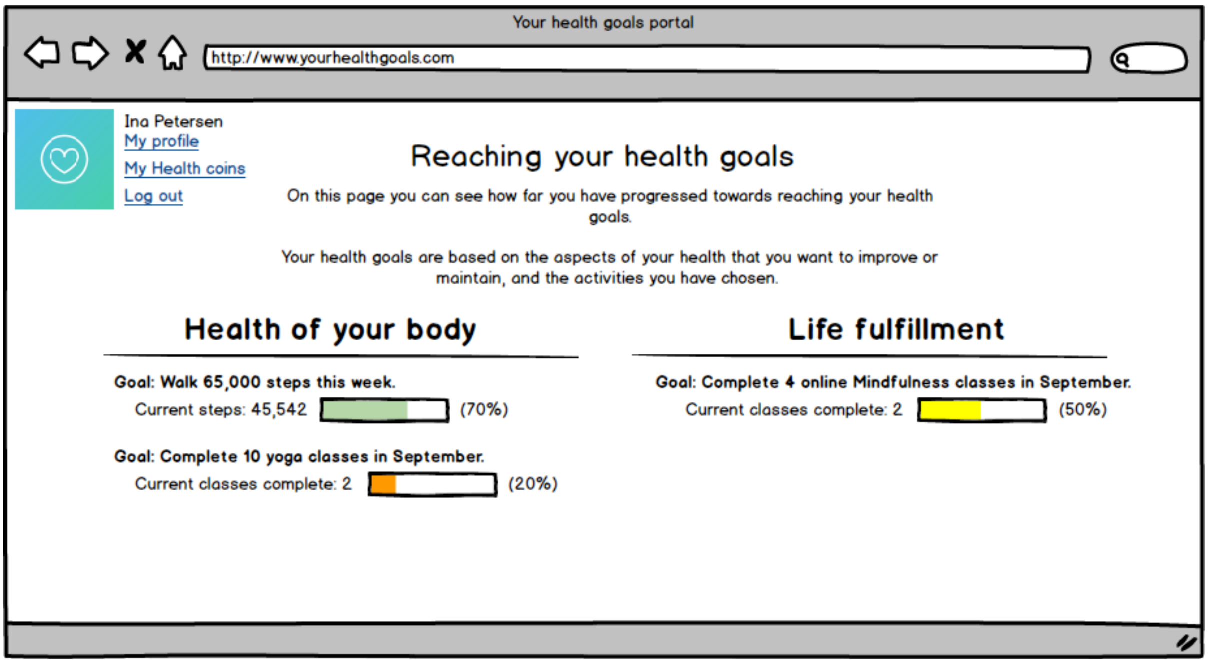

Feature 2: Social competition

On Yourhealthgoals.com we can show you how healthy you are acting, in comparison to your friends, colleagues, or other people that are like you (in terms of age, residence, etc.)

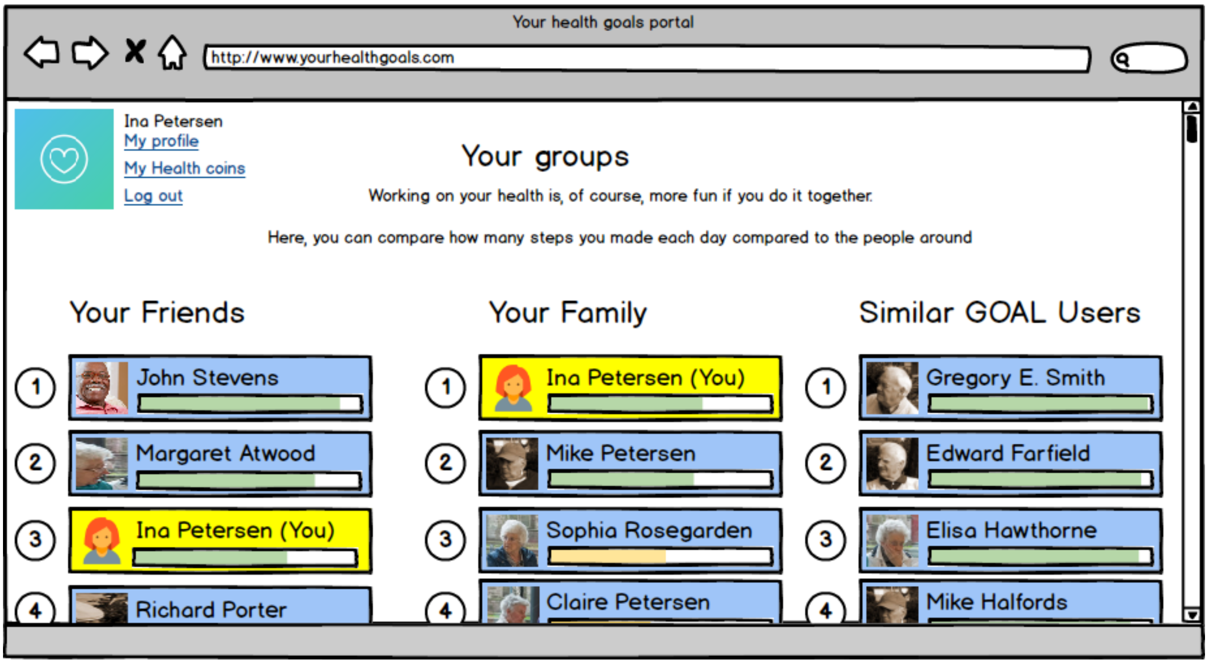

Feature 3: Social support

On Yourhealthgoals.com we can help you to find people like you, or people you already know. This way, you can become friends via the website, exchange experiences or encourage each other while living a healthy life.

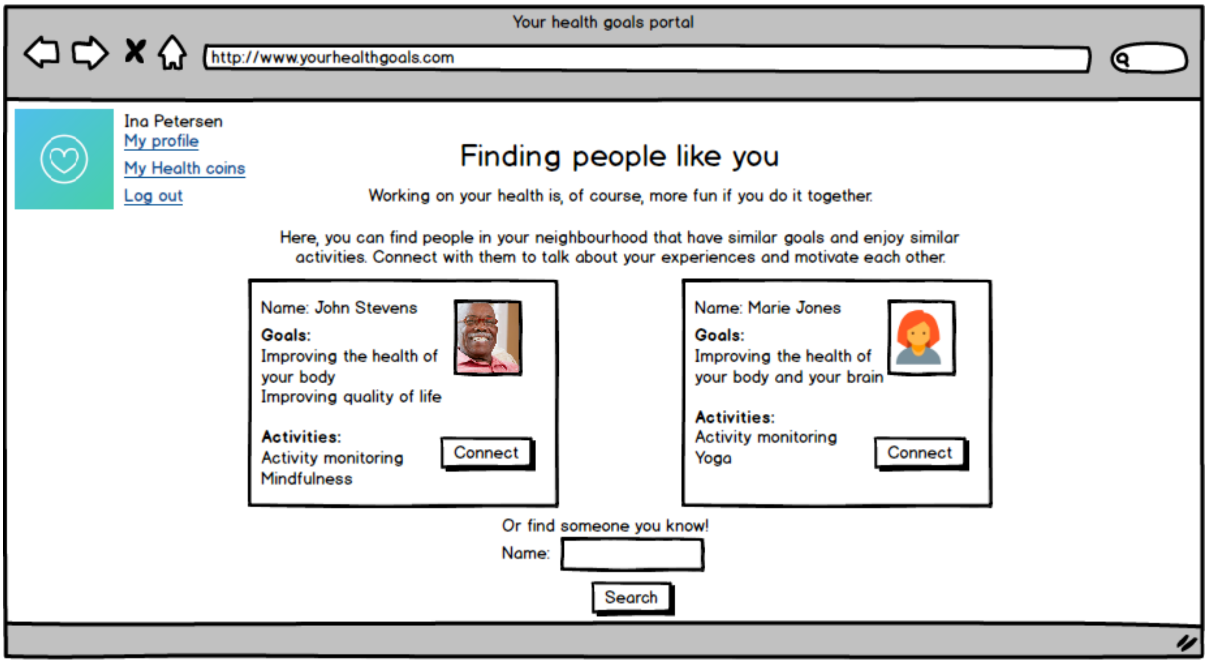

Feature 4: Reminders

Via Yourhealthgoals.com we can remind you to stay healthy. We can do this by reminding you to do exercises to keep your brain in shape, to go out for a walk, or to do something nice with your friends.

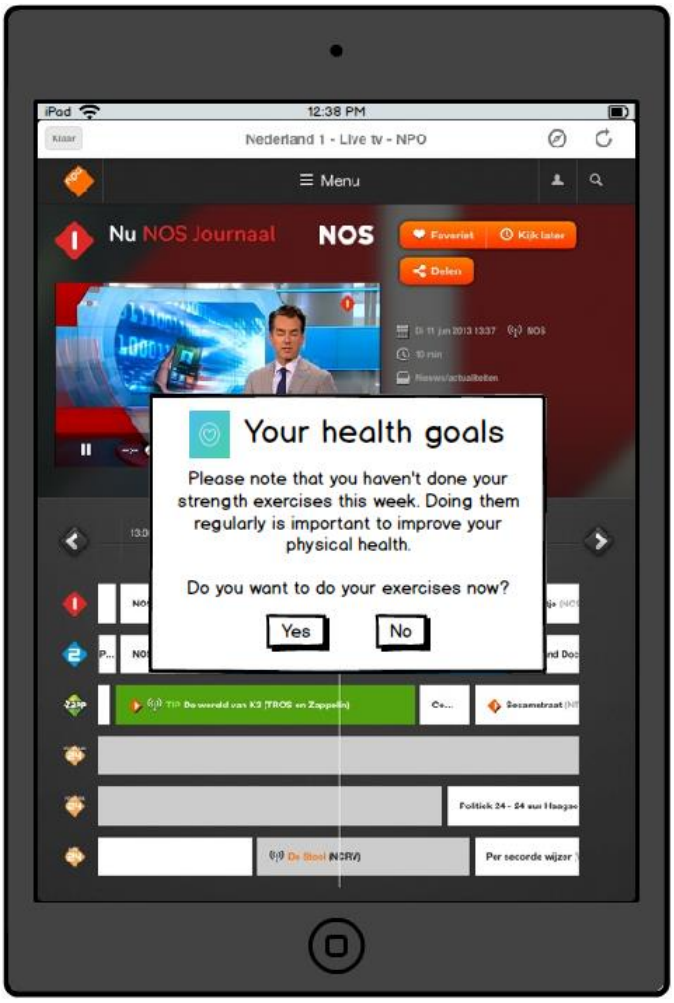

Feature 5: Fear appeals

On Yourhealthgoals.com we can calculate for you what your real age is. We can do this by looking at how active you are and how healthy you live.

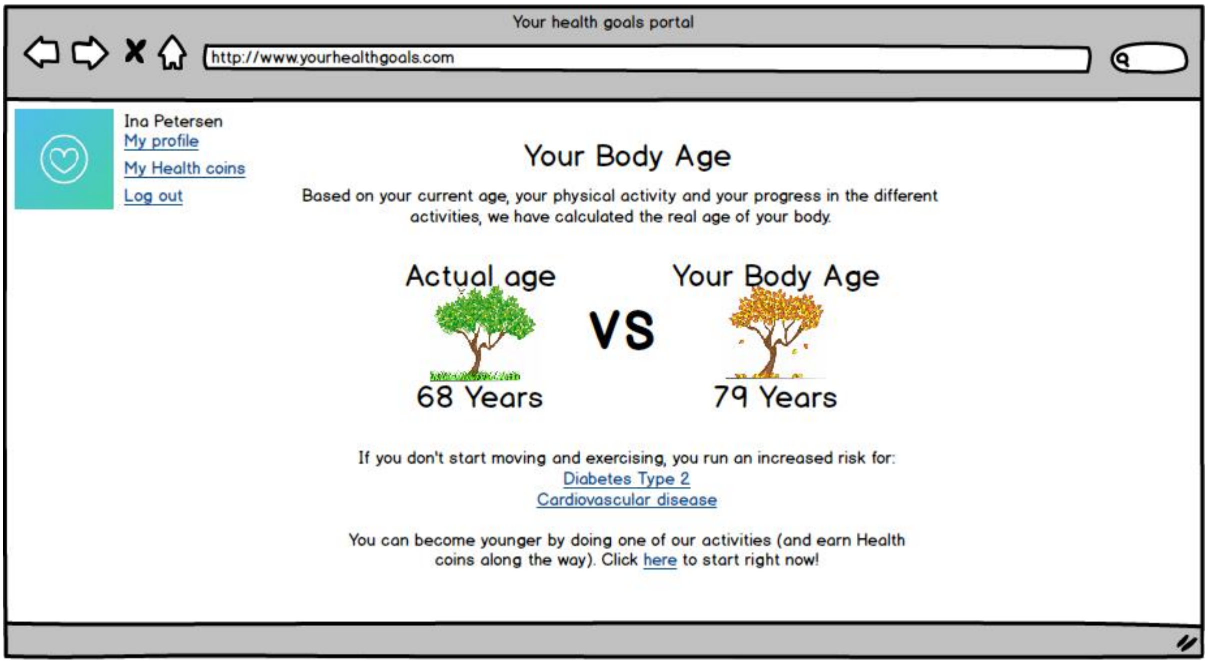

Feature 6: Self goal setting

On Yourhealthgoals.com we can help you to achieve your goals. You can set a goal that you like yourself (like 6,400 steps on a day). Then, you can see how close you are towards reaching the goal.

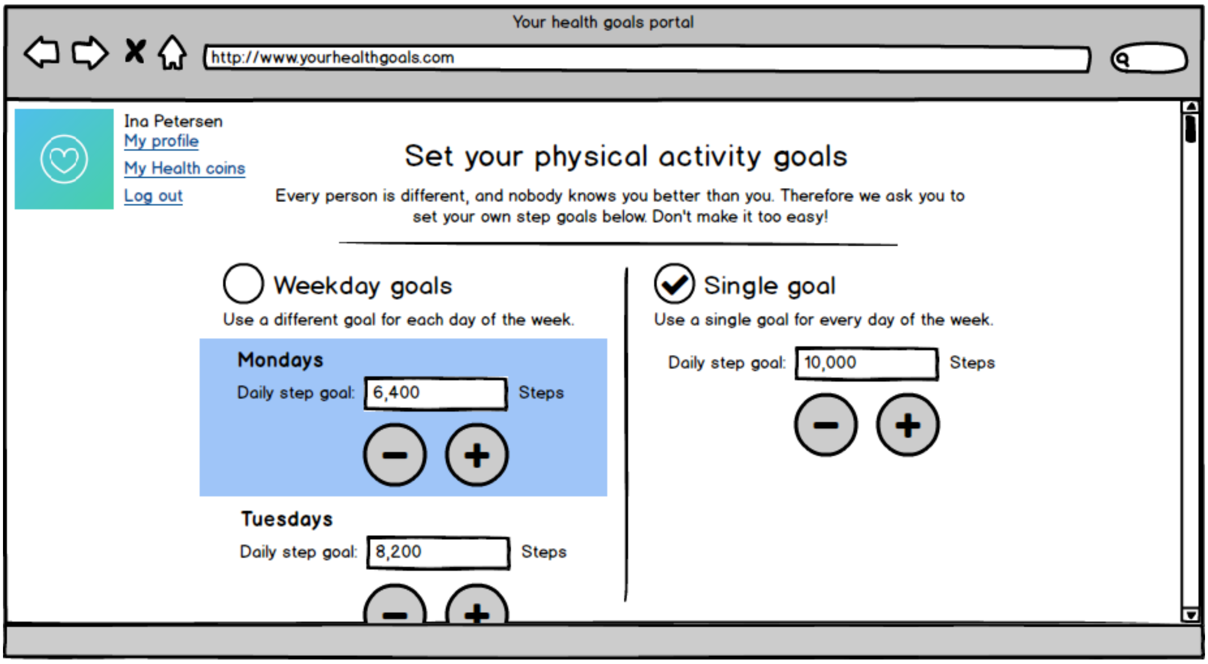

Feature 7: Automatic goal setting

On Yourhealthgoals.com we can help you to achieve your goals by setting them automatically for you. The website then decides what is a good goal for you. For this, the website uses information on how much you have been active, for example.

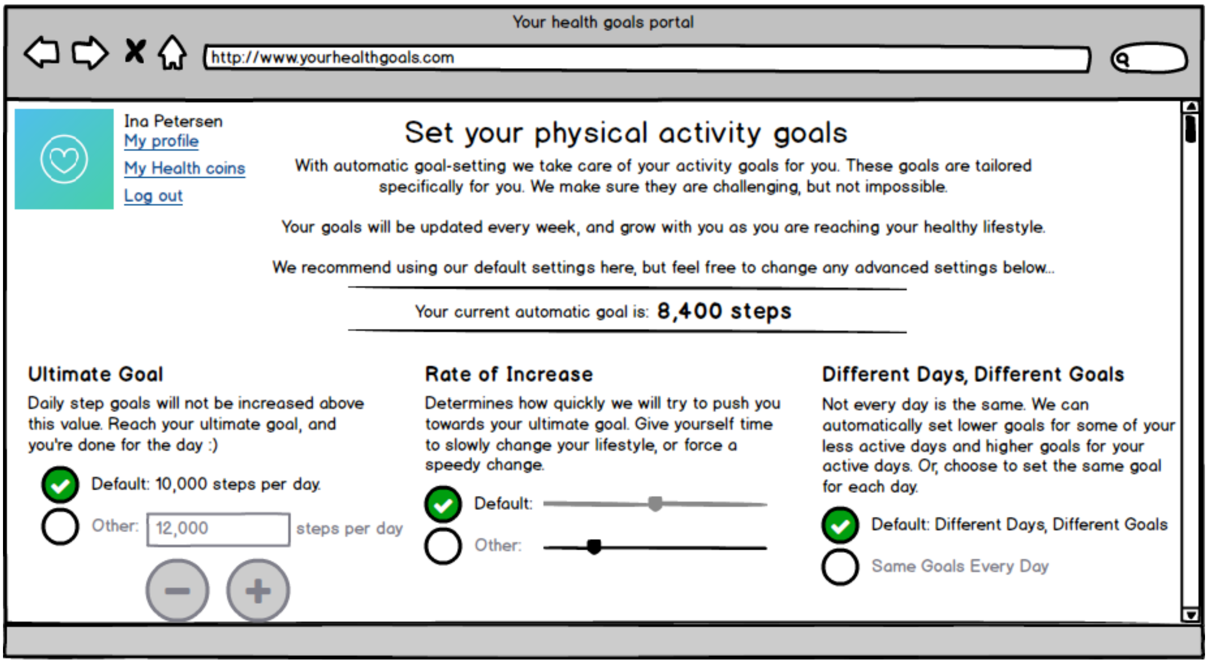

Feature 8: Rewards, compliments

On Yourhealthgoals.com you can receive rewards for achieving your personal goals. If you want to be more physically active, for example, you will get a reward when you have cycled 10 or 15 kilometres.

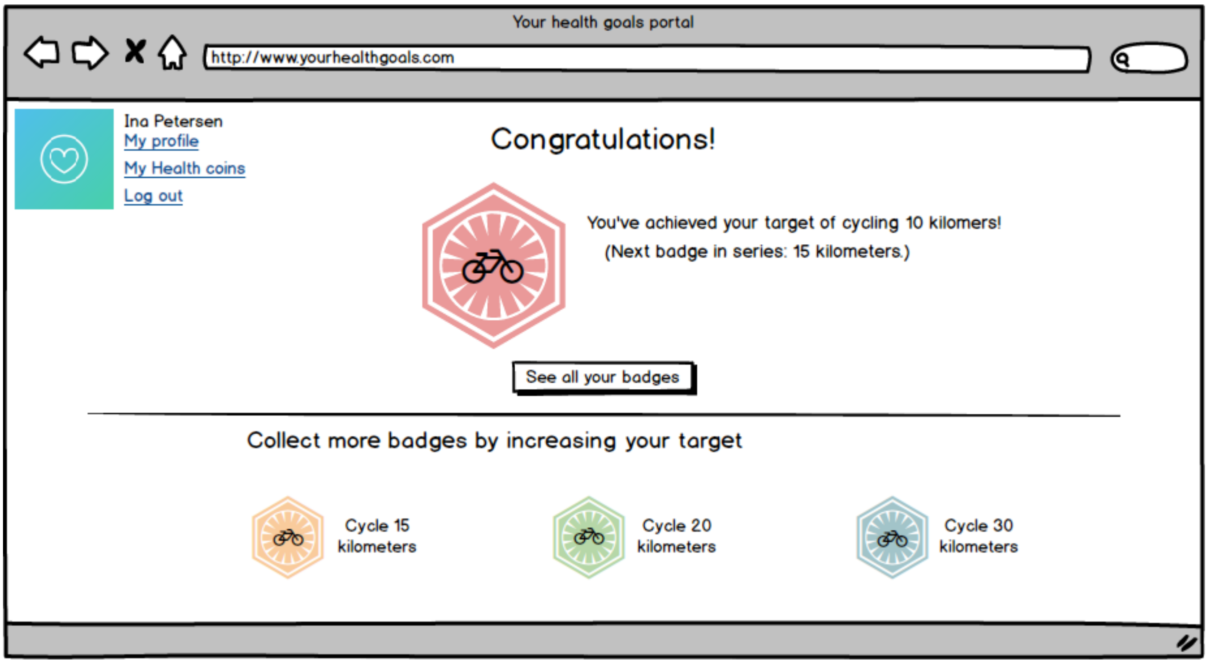

Feature 9: Rewards, monetary

On Yourhealthgoals.com you can collect coins when achieving your health goals. Let’s say that you have to do 8,000 steps on a day, then you will receive points when you have done so. You can then exchange these points for a discount in stores.

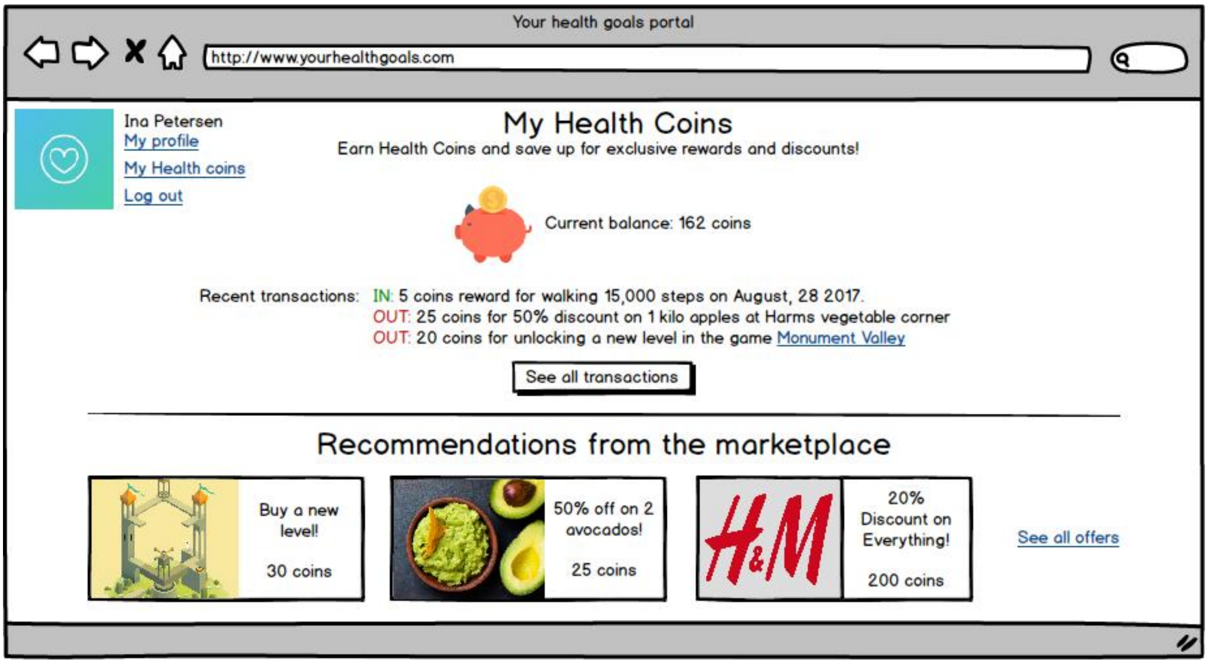

Feature 10: Health education

Via Yourhealthgoals.com we can give you information about the consequences of an unhealthy life. We can also tell you how to prevent getting ill.

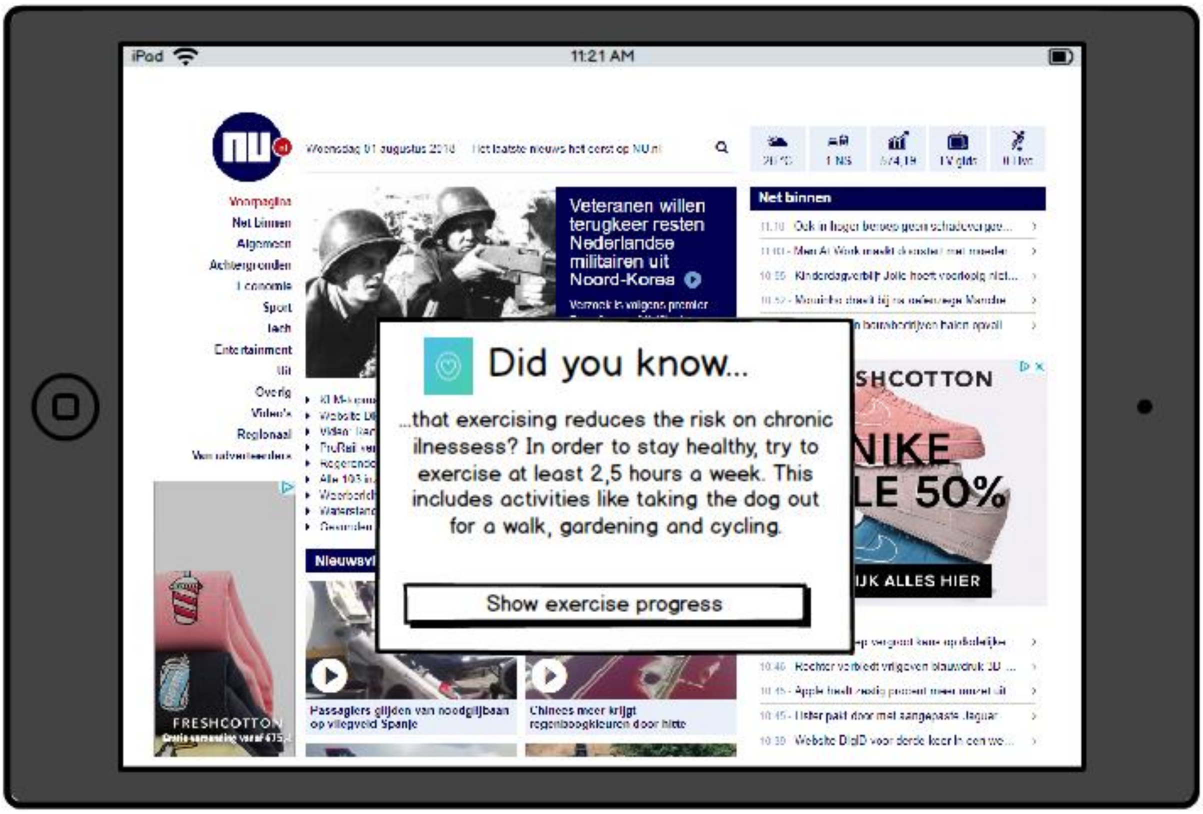

Feature 11: Implementation intentions

Via Yourhealthgoals.com you can plan for yourself what you want to do in the coming week. This way, you can plan, for example, that you will go for 30 minutes of swimming on Monday and 30 minutes of walking on Friday. The website will then remind you to do so.

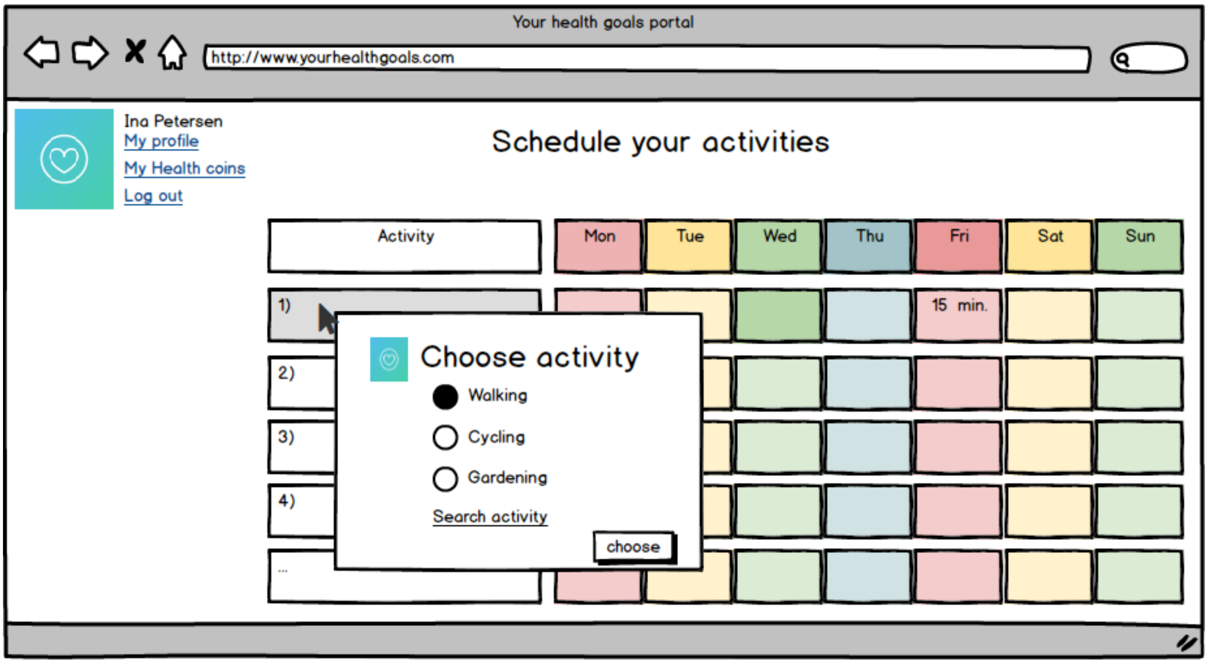

Supplement: Multimedia Appendix 1 [file jmir_v21i9e11759_app1.pdf]
