# Supplementary material for: Tailoring Persuasive Electronic Health Strategies for Older Adults on the Basis of Personal Motivation: Web-Based Survey Study
Source: J Med Internet Res. 2019 Sep 6;21(9):e11759. doi: 10.2196/11759 (PMC6788334; doi:10.2196/11759)
Supplement: Multimedia Appendix 2 [file jmir_v21i9e11759_app2.pdf]

Multimedia Appendix 2:

Final survey for classifying older adults

Intrinsic motivation

Because I like to learn more about healthy living

Because I like to discover new ways to lead a healthier life

Because I think it's very interesting to learn how to live a healthier life

Because I think it is a good way to develop my strong suits

Because I think it's one of the best ways to develop other sides of myself

External regulation

Because the people that are important to me would be angry at me if I didn't

Because I would then be appreciated by the people I know

Because I think other would disapprove of me if I didn't

So that I get compliments from others

A-motivation

I used to have good reasons to live a healthy life, but lately I'm doubting whether or not to continue with that

I don't think that living a healthy life really is something for me

Notes

All items are to be accompanied by a seven-point Likert scale, ranging from fully disagree, to fully agree.
